# Supplementary material for: FERN – a Java framework for stochastic simulation and evaluation of reaction networks
Source: BMC Bioinformatics. 2008 Aug 29;9:356. doi: 10.1186/1471-2105-9-356 (PMC2553347; doi:10.1186/1471-2105-9-356)
Supplement: Additional file 1 — FERN distribution, Version 1.3. This archive contains the FERN source code and binaries as well as documentation and example models in FernML and SBML. [file 1471-2105-9-356-S1.zip › fern/doc/javadoc/fern/tools/gnuplot/Axes.html]

Axes


---


|  |  |  |
| --- | --- | --- |
| **Overview** \| **Package** \| **Class** \| **Use** \| **Tree** \| **Deprecated** \| **Index** \| **Help** | |  |
| **PREV CLASS**   **NEXT CLASS** | **FRAMES**    **NO FRAMES**     **All Classes** |  |
| SUMMARY: NESTED \| FIELD \| CONSTR \| METHOD | DETAIL: FIELD \| CONSTR \| METHOD |  |


---


## fern.tools.gnuplot Class Axes

```
java.lang.Object
  fern.tools.gnuplot.Axes
```

**All Implemented Interfaces:**: Iterable<String>

**Direct Known Subclasses:**: ArrayMatrixAxes, CollectionAxes

---

``` public abstract class Axes extends Object implements Iterable<String> ```

Contains the data columns for `GnuPlot`. `GnuPlot` has a list of
`Axes` objects and assumes that each of these `Axes` object has one
x column (the first one) and n value columns.

The main access point to the data is the `Iterable` interface which yields
the tab separated values of each columns row by row.

It is possible to add other `Axes` object to an `Axes` object. So
If you call the `Iterable.iterator()`, all of the columns of each attached `Axes`  object will be yielded. Hence attached `Axes` objects must have the
same number of rows (if they don't, an `IllegalArgumentException` will be thrown).

This class also manages labels and styles for each column. If you specify either of them,
then either the length must be equal to the number of columns or 1 shorter (then the
`Axes` objects knows that its first column is an x column).

The content of labels is directly passed to the `plot` command as `title "label"`,
the content of styles also is attached to the corresponding place in the `plot`
command (e.g. styles[i]="with linespoints").

**Author:**
:   Florian Erhard

---

**Constructor Summary**

`Axes()`


| **Method Summary** | |
| --- | --- |
| `void` | `addAxes(Axes axes)`             Attaches an `Axes` object to this one. |
| `void` | `applyDefaultStyle(String defaultStyle)`             Sets the style of each column to the default style except for columns that already have a style. |
| `protected  Iterator<String>` | `getAdditionalAxesIterator()`             Iterator for the attached `Axes` objects. |
| `String` | `getLabel(int col)`             Gets the label for a column. |
| `protected  int` | `getNumAdditionalColumns()`             Gets the number of columns of the attached `Axes` objects. |
| `abstract  int` | `getNumColumns()`             Gets the number of columns. |
| `abstract  int` | `getNumRows()`             Gets the number of rows. |
| `String` | `getStyle(int col)`             Gets the style for a column. |
| `protected  void` | `loadLabelsAndLineStyles(String[] labels, String[] styles)`             Adds new labels and styles to the axes object. |
| `void` | `setLabel(int col, String label)`             Sets the label for a column. |
| `void` | `setStyle(int col, String style)`             Sets the style for a column. |

**Methods inherited from class java.lang.Object**

`clone, equals, finalize, getClass, hashCode, notify, notifyAll, toString, wait, wait, wait`

**Methods inherited from interface java.lang.Iterable**

`iterator`

**Constructor Detail**

### Axes

```
public Axes()
```


**Method Detail**

### loadLabelsAndLineStyles

```
protected void loadLabelsAndLineStyles(String[] labels,
                                       String[] styles)
```

:   Adds new labels and styles to the axes object. If either of them is not `null`
    then the length must be equal to the number of columns or 1 shorter.

    :   **Parameters:**: `labels` - labels for the columns: `styles` - styles for the columns

---


### addAxes

```
public void addAxes(Axes axes)
```

:   Attaches an `Axes` object to this one. It cannot have an `Axes`
    object with an x axis (which means that the labels/styles array is 1 shorter then the number
    of columns).

    Additionally the numbers of rows must be equal.

    :   **Parameters:**: `axes` - the `Axes` object to attach

---


### getNumColumns

```
public abstract int getNumColumns()
```

:   Gets the number of columns. The number of the attached `Axes`
    object is also included.

    :   **Returns:**: number of columns.

---


### getNumRows

```
public abstract int getNumRows()
```

:   Gets the number of rows.

    :   **Returns:**: number of rows.

---


### setLabel

```
public void setLabel(int col,
                     String label)
```

:   Sets the label for a column. If this `Axes` object has a x axis, the label
    of the 0th column cannot be set (because it is the x axis).

    :   **Parameters:**: `col` - zero based index of the column: `label` - label for the column

---


### getLabel

```
public String getLabel(int col)
```

:   Gets the label for a column. If this `Axes` object has a x axis, the label
    of the 0th column cannot be retrieved (because it is the x axis).

    :   **Parameters:**: `col` - zero based index of the column **Returns:**: label for the column

---


### setStyle

```
public void setStyle(int col,
                     String style)
```

:   Sets the style for a column. If this `Axes` object has a x axis, the style
    of the 0th column cannot be set (because it is the x axis).

    :   **Parameters:**: `col` - zero based index of the column: `style` - style for the column

---


### getStyle

```
public String getStyle(int col)
```

:   Gets the style for a column. If this `Axes` object has a x axis, the style
    of the 0th column cannot be retrieved (because it is the x axis).

    :   **Parameters:**: `col` - zero based index of the column style style for the column

---


### applyDefaultStyle

```
public void applyDefaultStyle(String defaultStyle)
```

:   Sets the style of each column to the default style except for columns that already
    have a style.

    :   **Parameters:**: `defaultStyle` - the default style to set

---


### getNumAdditionalColumns

```
protected int getNumAdditionalColumns()
```

:   Gets the number of columns of the attached `Axes` objects.

    :   **Returns:**: number of attached columns

---


### getAdditionalAxesIterator

```
protected Iterator<String> getAdditionalAxesIterator()
```

:   Iterator for the attached `Axes` objects.

    :   **Returns:**: iterator


---


|  |  |  |
| --- | --- | --- |
| **Overview** \| **Package** \| **Class** \| **Use** \| **Tree** \| **Deprecated** \| **Index** \| **Help** | |  |
| **PREV CLASS**   **NEXT CLASS** | **FRAMES**    **NO FRAMES**     **All Classes** |  |
| SUMMARY: NESTED \| FIELD \| CONSTR \| METHOD | DETAIL: FIELD \| CONSTR \| METHOD |  |


---
